# Development of Chitosan Scaffolds with Enhanced Mechanical Properties for Intestinal Tissue Engineering Applications

**DOI:** 10.3390/jfb6040999

**Published:** 2015-10-13

**Authors:** Elie Zakhem, Khalil N. Bitar

**Affiliations:** 1 Wake Forest Institute for Regenerative Medicine, Wake Forest School of Medicine, Winston-Salem, NC 27101, USA; E-Mail: ezakhem@wakehealth.edu; 2 Department of Molecular Medicine and Translational Science, Wake Forest School of Medicine, Winston Salem, NC 27101, USA; 3 Virginia Tech-Wake Forest School of Biomedical Engineering and Sciences, Winston-Salem, NC 27101, USA

**Keywords:** chitosan, tubular scaffold, fibers, mechanical properties, freeze/dry, extrusion/gelation

## Abstract

Massive resections of segments of the gastrointestinal (GI) tract lead to intestinal discontinuity. Functional tubular replacements are needed. Different scaffolds were designed for intestinal tissue engineering application. However, none of the studies have evaluated the mechanical properties of the scaffolds. We have previously shown the biocompatibility of chitosan as a natural material in intestinal tissue engineering. Our scaffolds demonstrated weak mechanical properties. In this study, we enhanced the mechanical strength of the scaffolds with the use of chitosan fibers. Chitosan fibers were circumferentially-aligned around the tubular chitosan scaffolds either from the luminal side or from the outer side or both. Tensile strength, tensile strain, and Young’s modulus were significantly increased in the scaffolds with fibers when compared with scaffolds without fibers. Burst pressure was also increased. The biocompatibility of the scaffolds was maintained as demonstrated by the adhesion of smooth muscle cells around the different kinds of scaffolds. The chitosan scaffolds with fibers provided a better candidate for intestinal tissue engineering. The novelty of this study was in the design of the fibers in a specific alignment and their incorporation within the scaffolds.

## 1. Introduction

Diseases of the gastrointestinal tract including short bowel syndrome result from massive resections of the intestine [1]. Patients usually suffer from malabsorption and malnutrition which is associated with high mortality and morbidity rates [2]. One of the treatment options includes intestinal transplantation but it is limited by many factors such as immuno-rejection of the graft, shortage of donor organs, and the size of the graft to be transplanted [3,4]. Another option for patients is total parenteral nutrition [5]. This is associated with complications related to the catheter and liver diseases [6]. Therefore, engineered intestinal replacements offer an alternative promising solution. 

A wide range of materials exists for tissue engineering applications; natural, synthetic or a hybrid of the two. Previously, synthetic biomaterials polyglycolic acid (PGA) and polycaprolactone (PCL) were evaluated for intestinal tissue engineering applications [7,8]. Even though the polymers supported the regeneration of intestinal segments, the mechanical properties of the scaffolds were not evaluated. Collagen, as a natural material, was also evaluated for its potential in intestinal tissue engineering [9,10]. Collagen alone has weak mechanical properties which makes it unsuitable for intestinal tissue engineering applications. One of the limitations of using collagen alone as a scaffold is its fast degradation *in vivo* due to its low mechanical properties.

Chitosan is another natural material that is widely used in different tissue engineering applications [11]. Chitosan is a natural material derived from chitin with various degrees of deacetylation. Structurally, chitosan is a linear polysaccharide of 2-amino-2-deoxy-β-D-glucopyranose units linked by (1→4)-β-glycosidic bonds. The advantage of using chitosan in tissue engineering is its biocompatibility *in vivo*, its controllable degradation rate and mechanical properties. One of the approaches to modulate the mechanical properties of chitosan scaffolds involves modifying the manufacturing process [12]. Chitosan fibers have been recently reported to enhance the mechanical properties of chitosan scaffolds. 

We have previously demonstrated the biocompatibility of chitosan scaffolds in intestinal tissue engineering [13,14]. Smooth muscle and enteric neurons maintained their phenotype and functionality around chitosan scaffolds. In our previous studies, we engineered porous tubular chitosan-collagen scaffolds using the freeze/dry method. The mechanical properties of the porous tubular scaffolds did not match those of native intestines. The scaffolds failed to maintain their luminal patency. The aim of this study was to engineer circumferentially-aligned chitosan fibers and to incorporate them into the tubular scaffolds as a way to enhance the mechanical properties. We demonstrated that chitosan fibers circumferentially embedded in the tubular scaffolds have higher Young’s Modulus and burst strength than tubular chitosan scaffolds without fibers.

## 2. Materials and Methods

### 2.1. Reagents

Medium molecular weight chitosan (75%–85% deacetylation) was purchased from Sigma-Aldrich (St. Louis, MO, USA). Type I collagen was purchased from BD Biosciences.

### 2.2. Chitosan Fibers Preparation

A 2% w/v chitosan solution was prepared by dissolution in 0.2 M acetic acid. Chitosan fibers were formed using the extrusion/gelation method as described previously [15]. Chitosan solution was extruded using a syringe pump through a catheter into a flask containing 10 wt % ammonia solution. Fibers were allowed to neutralize in the ammonia solution and then dried at room temperature in different forms as described below. 

### 2.3. Scaffolds Preparation

Four different kinds of scaffolds were engineered, all having the same basic components (chitosan and collagen). A 2% w/v chitosan solution was prepared by dissolution in 0.2 M acetic acid. A 0.1 mg/mL collagen type I solution was prepared by dissolution in acetic acid. Tubular chitosan-collagen scaffolds were prepared using the freeze/dry method as described previously [14]. Briefly, the 2% w/v chitosan solution was mixed with 0.1 mg/mL rat tail type I collagen in a volume ratio of 1:1. A custom made mold was used to prepare the scaffolds. The mold consisted of an outer cylindrical tube of 0.7 cm diameter. An additional tube of 0.3 cm diameter was inserted in the center of the outer tube to create the inner lumen of the scaffold.

a) Porous scaffolds

The chitosan-collagen mixture was poured into the space between the two tubes of the mold. The composite was then frozen at −80 °C for 3 h and then lyophilized for 24 h.

b) Scaffolds with inner fibers

Chitosan fibers were consistently wrapped circumferentially, attached to each other, around the inner tube of the mold and allowed to dry at room temperature. Following drying, the tube was inserted into the center of the mold. The same chitosan-collagen mix was then poured into the space between the two tubes of the mold. The mold was then frozen at −80 °C for 3 h and then lyophilized for 24 h.

c) Scaffolds with outer fibers

The chitosan-collagen solution was first poured into the space between the two tubes of the mold followed by freezing the sample at −80 °C for 3 h and then lyophilizing for 24 h. Following lyophilization, the prepared chitosan fibers were wrapped circumferentially, attached to each other, around the dried chitosan scaffold. The fibers were allowed to dry around the scaffold at room temperature in a similar manner as described above.

d) Fiber-sandwiched scaffolds

Fiber-sandwiched scaffolds were prepared as a combination of the two previously described scaffolds. Briefly, after preparing the scaffolds with inner fibers as described in (b), additional chitosan fibers were wrapped circumferentially around the lyophilized scaffolds and allowed to dry. After drying, the scaffolds were analyzed.

### 2.4. Characterization of the Fibers and Scaffolds

The chitosan fibers and scaffolds were observed under scanning electron microscopy. Chitosan fibers were allowed to dry at room temperature and used for imaging. Different types of lyophilized scaffolds were sectioned using a sharp scalpel. Fibers and cross sections of scaffolds were mounted on a stub with double sticky tape and colloidal graphite. Samples were then sputter-coated with gold prior to examination under scanning electron microscopy (SEM; Model S-2260N, Hitachi Co. Ltd., Tokyo, Japan).

### 2.5. Tensile Properties of the Scaffolds

Lyophilized scaffolds were neutralized in 0.2 M NaOH and then washed with phosphate buffer saline (PBS) at room temperature. Scaffolds were cut into tubular structures of 30 mm length, 30 mm inner diameter and 0.2 mm thickness. Tensile properties were measured using a uniaxial load test machine (Model # 5544, Instron Corporation, Issaquah, WA, USA) equipped with a maximum 2 kN load cell at a crosshead speed of 0.5 mm/s. Measurements of tensile strength, Young’s modulus and elongation at break were obtained from stress-strain curves. Native rat intestine was used as control.

### 2.6. Burst Pressure Strength

The burst pressure strength of the different kinds of scaffolds was measured by increasing the luminal pressure inside the scaffolds until failure. The test was performed using a pressure transducer catheter which was inserted inside the scaffolds. The luminal pressure was gradually increased using a pressure syringe until failure of the scaffolds occurred. The pressures were then recorded.

### 2.7. Cell Adhesion and Alignment on the Scaffolds

Smooth muscle sheets were engineered following our previously published methods. Briefly, intestine-derived smooth muscle cells were isolated by collagenase digestion. Cells were then grown on wavy molds with longitudinal grooves. Following cell alignment along the grooves, a collagen gel was then overlaid on top of the aligned smooth muscle. A smooth muscle sheet formed and was lifted off the plate. The sheets were then wrapped circumferentially around the different tubular scaffolds and left in culture *in vitro* for 14 days. At the end of the culture period, the scaffolds were fixed in 3.7% formaldehyde and processed. Cross sections of 6 µm thickness were stained with α-smooth muscle actin (Sigma-Aldrich, Saint Louis, MO, USA) and DAPI to evaluate maintenance of cell phenotype and alignment on the scaffolds.

### 2.8. Statistical Analysis

The differences in tensile strength, tensile strain, Young’s modulus, and burst strength between the different types of scaffolds and the native tissue were evaluated using ANOVA. Values reported are mean ± SEM. Differences were considered statistically significant for *p* < 0.05.

## 3. Results

### 3.1. Characterization of the Fibers and Scaffolds

**Figure 1:**
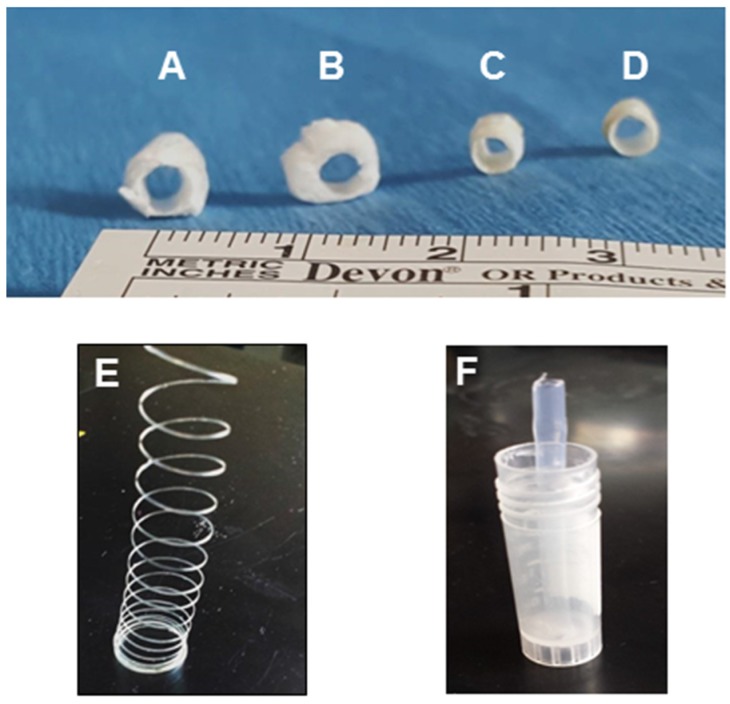
Representative images of the tubular scaffold (**A**) without fibers; (**B**) with inner fibers; (**C**) with outer fibers; and (**D**) with inner and outer fibers (fiber sandwiched scaffold). (**E**) Circumferentially aligned chitosan fibers were engineered using the extrusion/gelation method. (**F**) The mold for scaffolds consisted of an outer tube and an inner tube to create the lumen of the scaffold.

**Figure 2:**
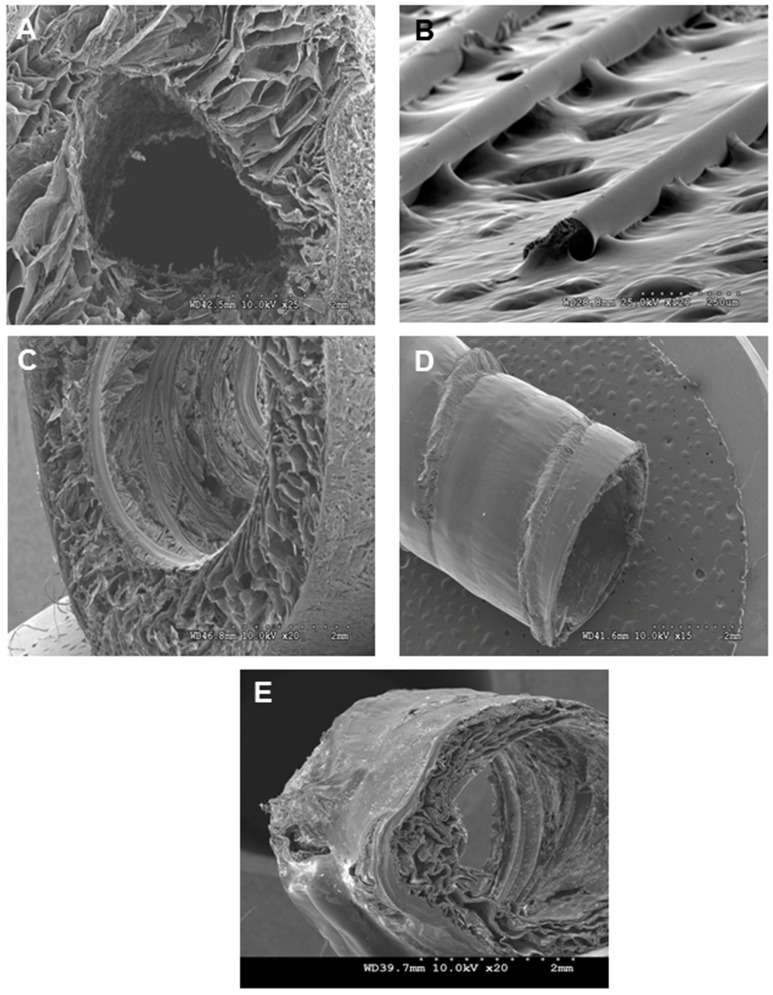
Representative SEM images of the tubular scaffold (**A**) without fibers; (**B**) the fibers; (**C**) scaffold with inner fibers; (**D**) scaffold with outer fibers; and (**E**) with inner and outer fibers.

**Figure 3:**
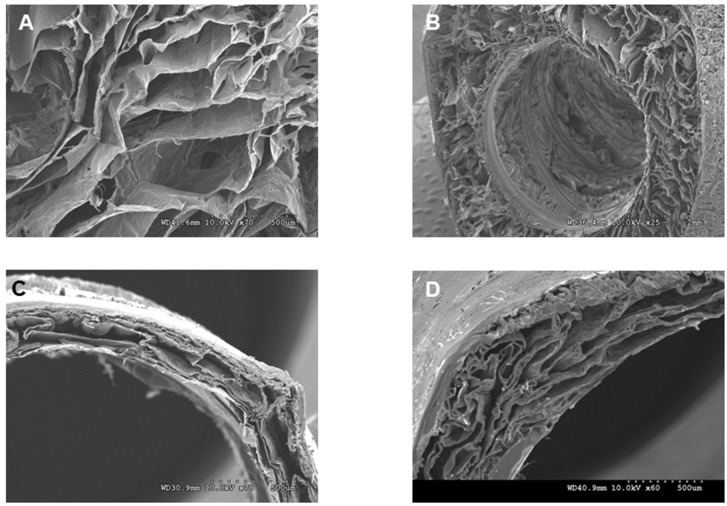
SEM of the different scaffolds shows the morphology of the pores. (**A**) scaffold without fibers; (**B**) scaffold with inner fibers; (**C**) scaffold with outer fibers and (**D**) fiber sandwiched scaffold.

Tubular chitosan scaffolds with luminal opening were prepared following the freeze/dry method. The scaffolds were 3 cm in length and 0.3 cm internal diameter. We developed a technique to form continuous, circumferentially-aligned, chitosan fibers using the extrusion/gelation method. Representative images of the mold, the scaffolds and the fibers are shown in Figure 1. Representative SEM images of the fibers and the different scaffolds are illustrated in Figure 2. The fibers appeared to be incorporated within the scaffold, either from the luminal side or from the outer side of the tube. The scaffolds without fibers had a wall thickness of 1.7 ± 0.1 mm (Figure 2A). Those scaffolds did not maintain their luminal patency in wet conditions. The fibers had a diameter of 100 µm (Figure 2B). For the scaffolds with inner fibers, the inner tube of the mold was used to wrap the fibers around it. The fibers were then allowed to dry. The chitosan-collagen solution was poured into the annular space of the mold, frozen, and then lyophilized. The fibers became incorporated within the lumen of the scaffold (Figure 2C). The fibers were uniformly aligned around the inner tube. The distance between the inner fibers was 0.4 mm. The engineering process was consistent in that the fibers were uniformly distributed within the lumen of the scaffolds. The wall thickness of the scaffolds with inner fibers was 1.3 mm. For the chitosan scaffolds with outer fibers, the fibers were wrapped circumferentially around the scaffolds and allowed to dry (Figure 2D). The scaffolds shrunk and their wall thickness was 0.4 mm. The distance between the outer fibers was 2.9 mm. The fiber-sandwiched scaffolds had two layers of circumferentially aligned fibers; one layer on the outside and one layer on the inside. The scaffolds also shrunk with wall thickness of 0.4 mm (Figure 2E). Figure 3 shows an SEM of the porosity of the different scaffolds. A non-uniform morphology was seen, especially in the scaffolds with outer fibers and the fiber-sandwiched scaffolds. 

### 3.2. Mechanical Properties

The summary of the mechanical properties of the different kinds of scaffolds is presented in Table 1.

**Table 1:** Summary of the mechanical properties of the different kinds of scaffolds.

Samples	Tensile Stress (MPa)	Elongation at Break (%)	Young’s Modulus (MPa)	Burst Pressure (mmHg)
Native intestine	0.076 ± 0.007	230 ± 13	0.122 ± 0.01	–
Scaffolds without fibers	0.013 ± 0.003	124 ± 6	0.022 ± 0.004	715 ± 38
Scaffolds with inner fibers	0.065 ± 0.008	262 ± 35	0.117 ± 0.007	1327 ± 75
Scaffolds with outer fibers	0.071 ± 0.01	137 ± 10	0.101 ± 0.019	1276 ± 75
Fiber sandwiched scaffolds	0.243 ± 0.033	114 ± 9	0.392 ± 0.089	–

a) Tensile properties

**Figure 4:**
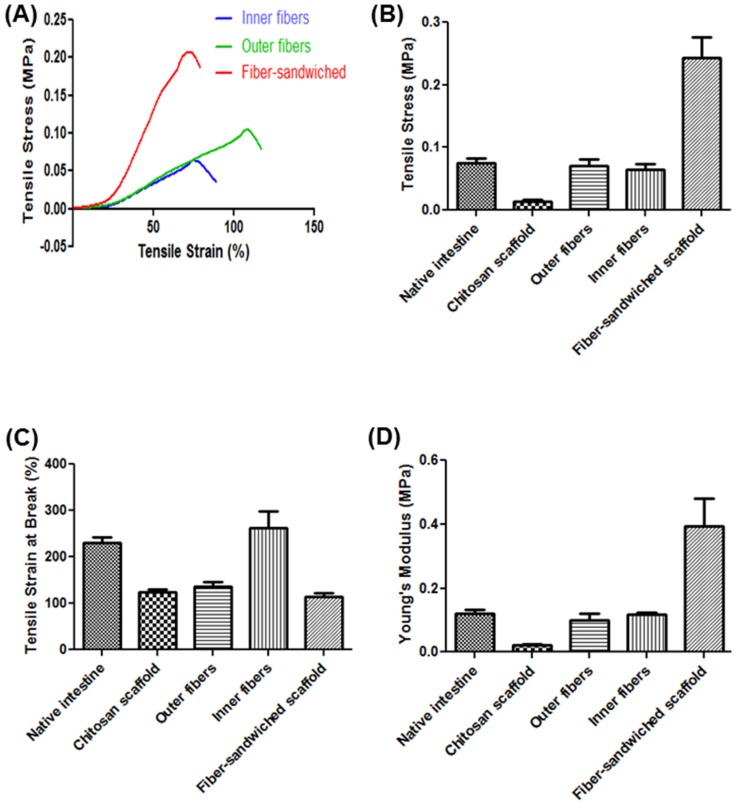
Tensile properties of the different kinds of scaffolds compared to native intestine: (**A**) Stress-strain curve; (**B**) Tensile stress; (**C**) Tensile strain at break, and (**D**) Young’s modulus.

Tensile properties of the different scaffolds and the native intestines were compared. The tensile properties were measured using the Instron machine which generated the stress-strain curve of the various scaffolds and tissues. Tensile strength, elongation at break, and Young’s modulus were obtained from stress strain curves (Figure 4A). The tensile stress of the scaffolds with inner or outer fibers was significantly increased when compared to the porous scaffolds without fibers and not significantly different from the native intestine (Figure 4B). However, the tensile stress of the fiber-sandwiched scaffolds was significantly higher from the rest of the scaffolds and the native intestine. The tensile stress of the porous chitosan scaffolds increased from 0.013 ± 0.003 MPa to 0.065 ± 0.008 MPa (inner fibers), 0.071 ± 0.01 MPa (outer fibers) and 0.243 ± 0.033 MPa (fiber-sandwiched scaffolds). Elongation at break of chitosan scaffolds with inner fibers (262% ± 35%) was significantly increased to match that of the native intestine (230% ± 13%). Elongation at break for the chitosan scaffolds without fibers (124% ± 6%), the chitosan scaffolds with outer fibers (137% ± 10%), and fiber-sandwiched scaffolds (114% ± 9%) were significantly lower than the native intestine and the scaffolds with inner fibers (Figure 4C). Additionally, the Young’s modulus of the chitosan scaffolds reinforced with inner fibers was increased to 0.117 ± 0.007 MPa and that of chitosan scaffolds with outer fibers increased to 0.101 ± 0.019 MPa. The values were not significantly different from the native intestine (0.122 ± 0.01 MPa). Young’s modulus of the chitosan scaffolds without fibers (0.022 ± 0.004 MPa) was significantly lower than that of the native intestine. Young’s modulus of the fiber-sandwiched scaffolds (0.392 ± 0.089 MPa) was significantly higher than the native intestine and all other scaffolds (Figure 4D). 

b) Burst pressure strength

**Figure 5:**
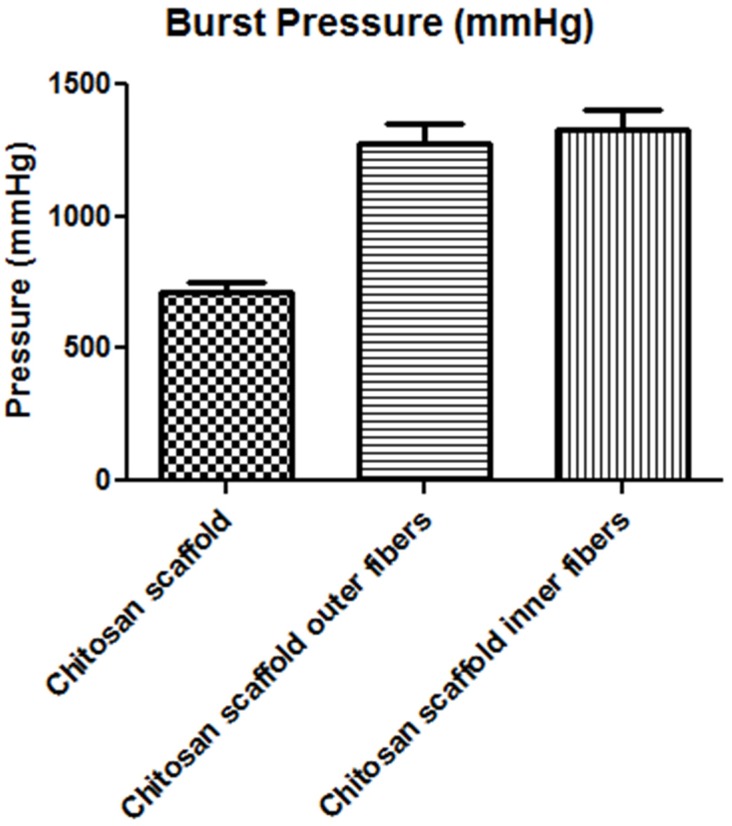
Burst pressure strength of different kinds of scaffolds.

Burst pressure strength test was performed to evaluate the maximum pressure that the scaffolds can withstand following increasing the luminal pressure of the tubular scaffolds. The pressures reported are the pressures at which the scaffolds failed (Figure 5). Both chitosan scaffolds with outer (1276 ± 75 mmHg) and inner (1327 ± 75 mmHg) fibers demonstrated significantly increased burst pressure strength when compared to porous chitosan scaffolds (715 ± 38 mmHg).

### 3.3. Cell Adhesion and Alignment on the Scaffolds

**Figure 6:**
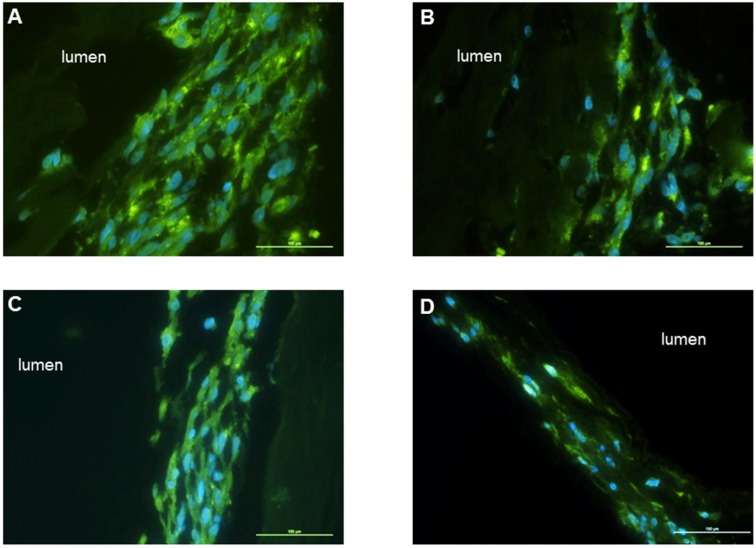
Different kinds of scaffolds were seeded with aligned smooth muscle sheets. Tissues stained positive for α-smooth muscle actin, indicating the maintenance of smooth muscle phenotype around all scaffolds. DAPI staining demonstrated maintenance of alignment of the cells around the scaffolds. (**A**) Scaffolds without fibers; (**B**) scaffolds with inner fibers; (**C**) scaffolds with outer fibers; and (**D**) fiber-sandwiched scaffolds. Scale bars 100 µm.

Smooth muscle cells aligned along the grooves of the molds. Five days following alignment, collagen gel was overlaid on top of the smooth muscle. Smooth muscle sheets were formed and wrapped circumferentially around the different tubular scaffolds. The tissues were left in culture for 14 days. Cross sections were stained with α-smooth muscle actin and DAPI to evaluate cell attachment and alignment (Figure 6). In all kinds of scaffolds, cells were shown to align circumferentially around the scaffold. Smooth muscle cells stained positive for α-smooth muscle actin indicating preservation of smooth muscle phenotype. DAPI staining showed maintenance of alignment of smooth muscle cells around the lumen of the tubular scaffolds.

## 4. Discussion

The selection of biomaterials is an essential factor in tissue engineering applications. When designing scaffolds, several factors must be taken into account, including biocompatibility of the scaffolds, biodegradability, and their mechanical properties. All these factors taken together result in engineered tissues with the characteristics required for successful outcomes. In intestinal tissue engineering, hollow tubular scaffolds are needed.

Even though the intestine is considered as a simple tubular structure, the complexity lies in the different types of cells that line the intestine. The mechanical properties of native gut tissues were previously tested. Given that there are different cell layers in the intestine, the contribution of each layer to the mechanical strength of the GI tract was an area of study. Previous reports have shown that the muscularis and the submucosal layers contribute the most to the mechanical strength of the intestinal wall [16,17]. Additionally, the physical properties of the luminal material of the GI tract change from liquid (small intestine) to solid (large intestine). In rats, the tensile strength increases from the proximal to the distal colon [18]. This is attributed to the increased in stress as the luminal content gets harder. The difference in mechanical strength of different segments of the gut is an essential factor when designing scaffolds for intestinal tissue engineering application. Another factor to take into account is the age of the patient. In the aging population, the connective tissue composition differs and the mechanical strength of the tissue decreases [19]. A successful scaffold must be compliant with the native tissue. Therefore, the mechanical properties of the engineered intestinal segments must match those of the native tissue and must be specific to the site of implantation and the age of the patients. 

Previous attempts to regenerate the intestine have focused on the source of cells seeded onto the scaffolds. There has been little focus on the mechanical properties of the engineered scaffolds. Studying the mechanics of the gastrointestinal tract helps in developing scaffolds suitable for the application. Natural and synthetic materials have been investigated as potential candidates in intestinal tissue engineering. Synthetic scaffolds can be consistently reproducible; however, these scaffolds lack the biomimetic properties which are essential for cell-scaffold interaction. Additionally, disadvantages include degradability byproducts and variation in mechanical properties which can be limiting in certain applications [20]. The synthetic polymer polyglycolic acid (PGA) was used as support for isolated organoid units [7]. The scaffolds were coated with collagen I as a way to enhance cell attachment. Ploy-ε-caprolactone (PCL) was also evaluated for its biocompatibility in intestinal tissue engineering [8]. Even though the mechanical properties can be modulated, the degradation rate of PCL is slow. This raises the problem of tissue remodeling *in vivo*. Even though the synthetic scaffolds showed promising results following their implantation in animal models, the mechanical properties of the scaffolds were not studied. Confirming the mechanical strength of the scaffolds is essential especially when it comes to scaling up the scaffolds for translational purposes.

On the other hand, the most common natural material collagen has been tested for intestinal tissue engineering application. Collagen has excellent biocompatibility, however, its low mechanical properties limits its use as the sole composition of the scaffold. We have previously-prepared scaffolds made of chitosan and collagen. We demonstrated the biocompatibility of chitosan-collagen composite scaffolds in intestinal tissue engineering [13,14]. The benefit of using chitosan as a component of our scaffold is its tunable mechanical properties, biocompatibility, controllable degradation rate and, finally, its ability to form porous scaffolds with different shapes. Our previous studies focused on engineering porous chitosan-collagen tubular scaffolds. We demonstrated that gut-derived smooth muscle cells and neurons maintained phenotype, morphology, and function when seeded onto the tubular scaffolds. However, the scaffolds failed to maintain their luminal patency. In this study, we investigated the possibility of increasing the mechanical strength of our scaffolds. We prepared chitosan fibers with a specific alignment. The advantage of our technique is the use of fibers made of the same material as the scaffold itself but in different physical property. Chitosan fibers have been previously used to enhance the mechanical properties of heart valve scaffolds [21]. In previous applications, small-cut fibers were randomly incorporated within the scaffolds and were able to improve the mechanical properties of the scaffolds. One disadvantage of this technique is the reproducibility of the manufacturing process. The randomness and the inconsistency in the amount of fibers incorporated within the scaffolds increase the variability of the mechanical properties. In our study, the fibers were consistently and uniformly prepared with a certain alignment. The fibers also improved the tensile properties of the tubular scaffolds. The scaffolds with fibers demonstrated mechanical strength similar to native intestine. The burst pressure is a measure of the maximal pressure that the scaffold can withstand before it fails and leaks. The burst pressure in intestinal tissue engineering application is important to ensure that the scaffold does not burst or leak due to luminal pressure. Our results indicated that the fiber-reinforced scaffolds have burst pressure that is almost 50% more than porous scaffolds without fibers.

In conclusion, we showed in this study that chitosan fibers can be developed with a specific and consistent pattern. We incorporated the fibers within tubular chitosan scaffolds. The mechanically reinforced scaffolds had higher tensile properties and burst pressure than scaffolds without fibers. In our case, we found that scaffolds with inner or outer fibers enhanced the mechanical properties of the tubular scaffolds to a similar extent. However, the scaffolds with outer fibers had decreased in wall thickness, which may be a limiting factor during surgical anastomosis with the native tissue and for cell infiltration during the regeneration process. Additionally, the fiber-sandwiched scaffolds have mechanical properties beyond the range of the native intestine, which make them non-suitable for our application. Future studies will be conducted to implant the scaffolds and evaluate their mechanical properties following implantation. This is important to ensure that the implants will maintain their integrity *in vivo* for long term success.
